# Decreased copy number of mitochondrial DNA: A potential diagnostic criterion for gastric cancer

**DOI:** 10.3892/ol.2013.1492

**Published:** 2013-07-25

**Authors:** SHI-LEI WEN, FENG ZHANG, SHI FENG

**Affiliations:** 1 Department of Human Anatomy, Academy of Preclinical and Forensic Medicine, West China Medicine College, Chengdu, Sichuan 610041, P.R. China; 2 West China Hospital, Sichuan University, Chengdu, Sichuan 610041, P.R. China; 3 Department of Thoracic and Cardiovascular Surgery, Huaihe Hospital of Henan University, Kaifeng, Henan 475000, P.R. China

**Keywords:** gastric cancer, mitochondrial DNA, copy number, clinicopathological stage

## Abstract

An alteration in the mitochondrial DNA (mtDNA) copy number has been detected in numerous human cancers. However, certain changes in the mtDNA copy number that occur during the initiation and progression of gastric cancer remain undetected. In the present study, using quantitative PCR analysis, the quantitative changes in mtDNA were observed during the initiation and progression of gastric cancer. Furthermore, the possible correlation between the changes in mtDNA and the clinicopathological stage were also investigated. However, the mechanism by which the change in mtDNA copy number occurs remains to be elucidated. Epigenetic changes are believed to play a significant role in regulating the mtDNA content. In order to determine whether there is a potential correlation between DNA methylation and mtDNA regulation, *in vitro* demethylation experiments were performed. Tumor tissues and corresponding non-cancerous tissues were surgically resected from 76 gastric cancer patients between 2010 and 2011. The results revealed that the average relative mtDNA copy numbers were 94.71±28.11 in the cancer tissues and 111.68±21.84 in the corresponding non-cancerous tissues (P<0.01). The quantitative changes in mtDNA demonstrated a significant decrease in gastric cancer, particularly in ill-defined stage III and IV cases, but had no association with gender. The mtDNA copy numbers demonstrated a marked increase (P<0.05) following demethylation treatment. The present results indicate that the mtDNA copy number plays a significant role during the progression of colorectal cancer, particularly during the late clinicopathological stages, and that the change in the mtDNA copy number may correlate with DNA methylation.

## Introduction

Gastric cancer is the fourth most common worldwide cancer and the second most common cause of cancer-related mortality in Asia and worldwide (1). In China, the incidence and mortality rates of gastric cancer have been recorded as 33.12 and 22.64, respectively, per 100,000 individuals (2). The prognosis of gastric cancer is extremely poor, with a 5-year survival rate of <30% (3). Human mitochondrial DNA (mtDNA) is a 16.6 kb closed circular duplex species of 16,569 bases, encoding 13 polypeptides that are involved in respiration and oxidative phosphorylation, in addition to 2 rRNAs and 22 tRNAs that are essential for protein synthesis in the mitochondria (4). In addition, mtDNA contains a non-coding region called the displacement loop (D-loop), which controls the replication and the transcription of mtDNA (4,5). DNA hypermethylation occurs at the CpG islands and is typically associated with gene silencing. Human mtDNA is present in high levels at 103–104 copies per cell (6). The mitochondria content and mtDNA copy number of an individual cell may vary with the type of cell and tissue, and the number may also change during cell differentiation and the aging process (7). Alterations have been reported in the mtDNA copy number in a variety of human carcinomas (8). However, alterations in the mtDNA content in gastric cancer and the corresponding non-tumorous gastric tissue remain elusive.

The present study determined the copy number of mtDNA in gastric cancer tissues and corresponding non-tumorous gastric tissues from male and female gastric cancer patients, in order to evaluate the alteration in the quantity of mtDNA and to reveal the correlation between the mtDNA copy number and the clinicopathological stages of gastric cancer. A demethylation experiment was performed on rat gastric mucosa cells to determine if DNA methylation and demethylation is involved in the alteration of the mtDNA copy number.

## Materials and methods

### Patients and specimens

A total of 76 gastric cancer tissues and corresponding non-cancerous tissues were surgically resected from patients at the West China Hospital (Sichuan University, Chengdu, Sichuan, China) between 2009 and 2011. This study was approved by the ethics committee of West China Hospital, Sichuan University (Chengdu, China). Informed consent was obtained from all the patients. Samples were obtained from 47 males and 29 females, aged 34–75 years (median, 51.9±8.7; Table I). All the specimens were fresh-frozen and maintained in liquid nitrogen immediately. The specimens of the tumor tissues were cut from the edge of the tumors and the corresponding non-cancerous tissues were collected from >5 cm away from the tumors. The investigation conforms to the principles outlined in the Declaration of Helsinki.

### DNA extraction and quantitative PCR analysis

A quantitative PCR method was used to determine the mtDNA copy number. The DNA specimens were prepared using the TIANamp Genomic DNA kit (DP304; Tiangen Biotech Co., Ltd., Beijing, China). The primers of β-actin (product length, 138 bp) were forward, 5′-CGGGAAATCGTGCGTGACAT-3′ and reverse, 5′-GAA GGAAGGCTGGAAGAGTG-3′. The PCR cycling conditions consisted of an activation step at 95°C for 10 min, followed by 40 cycles for 25 sec at 95°C, 1 min at 45°C and 20 sec at 72°C. The primers of mtDNA (product length, 487 bp) were forward, 5′-TACTCACCAGACGCCTCAACCG-3′ and reverse, 5′-TTA TCGGAATGGGAGGTGATTC-3′. The PCR cycling conditions consisted of an activation step at 95°C for 10 min, followed by 40 cycles for 25 sec at 95°C, 1 min at 35°C and 35 sec at 72°C. All PCRs were performed on a 7900HT Fast Real-Time PCR system (Applied Biosystems, Carlsbad, CA, USA) and each specimen was run in triplicate. The average of all three measurements was calculated. A negative control was included in each reaction.

### Primary rat gastric mucosa cell culture and demethylation experiment

Primary rat gastric mucosa cells were cultured as previously described (9). 5-Aza-2′-deoxycytidine (5-Aza; Sigma, St Louis, MA, USA) was used to induce mtDNA demethylation. Cells (5×10^5^) were seeded into 6-well plates in 2 ml medium. Following a 24-h incubation period, the medium was removed and the cells were incubated in 2 ml fresh medium containing a final concentration of 5 μM 5-Aza for 96 h. Following the treatment, the medium was removed and the cells were subjected to an additional 24-h incubation.

### Statistical analysis

The qualitative and quantitative changes in the mtDNA were analyzed using SPSS version 15.0 (SPSS, Inc., Chicago, IL, USA). The relative mtDNA copy numbers (mtDNA1/β-actin) of every specimen were calculated and Student’s t-test was used to analyze the difference in the mtDNA copy numbers between the tumor and the corresponding non-cancerous tissues and also the difference between genders. The quantitative data are expressed as the mean ± standard deviation.

## Results

### Decreased relative mtDNA copy number in gastric cancer

**Figure 1:**
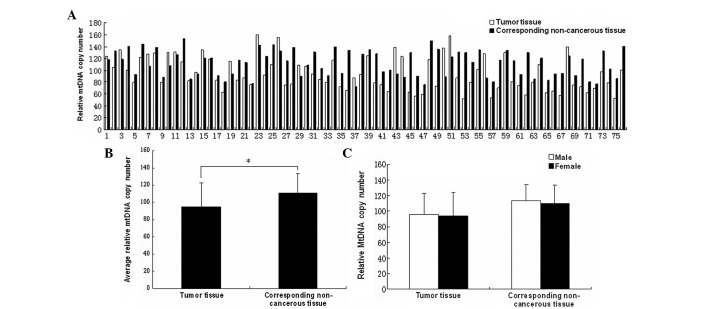
(A) Comparison of the relative mtDNA copy number between gastric cancer tissues and corresponding non-cancerous tissues of each case. (B) Comparison of the average relative mtDNA copy number between 76 gastric cancer tissues and the corresponding non-cancerous tissues. The mtDNA copy number of the tumor tissues was significantly higher than that of the corresponding non-cancerous tissues (^*^P<0.05). (C) Comparison of the average relative mtDNA copy numbers of 76 gastric cancer tissues and the corresponding non-cancerous tissues between genders. The mtDNA copy number of the tumor tissues showed no significant difference (P=0.621). In the corresponding non-cancerous tissues, there was also no significant difference (P=0.478). mtDNA, mitochondrial DNA

To identify the alteration in the mtDNA copy number in the gastric cancer tumor tissues, the mtDNA copy number was quantified in the 76 gastric cancer and corresponding non-cancerous stomach tissues. Following the statistical analysis, the results revealed that compared with the non-cancerous tissues, the relative mtDNA copy numbers of the tumor tissues were markedly decreased (Fig. 1B; P<0.05). The average relative mtDNA copy numbers were 94.71±28.11 in the tumor tissues and 111.67±21.84 in the corresponding non-cancerous tissues. No significant difference was noted between the male and female patients in the tumor and corresponding non-cancerous tissues (Fig. 1C; P>0.05).

### Correlation between relative mtDNA copy number and clinicopathological stages

**Figure 2:**
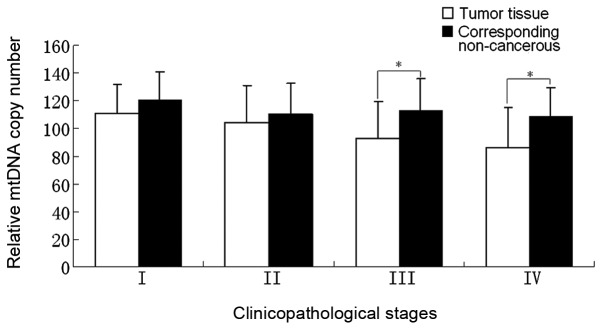
Average relative mtDNA copy number of gastric cancer of each clinicopathological stage I–IV. (^*^P<0.05). mtDNA, mitochondrial DNA.

The correlation between the clinicopathological stage and relative mtDNA copy number of 76 cancer cases was analyzed and the average mtDNA copy number of each stage of the tumor tissues and their corresponding non-cancerous tissues are shown in Table II. From stage I to stage IV, the relative mtDNA copy number of tumor tissues was lower than that of the corresponding non-cancerous tissues in each stage and these differences exhibited a marked difference in stages III and IV (P<0.05). While in stages I and II there were no marked differences (P>0.05; Fig. 2). Stages I and II were defined as group 1 and stages III and IV as group 2 (Table III). A marked difference was noted between groups 1 and 2 with regard to the relative mtDNA copy number between the tumor tissues (P<0.05). However, no significant difference was observed between the corresponding non-cancerous tissues (P=0.427).

### Demethylation treatment and increased mtDNA copy number

**Figure 3:**
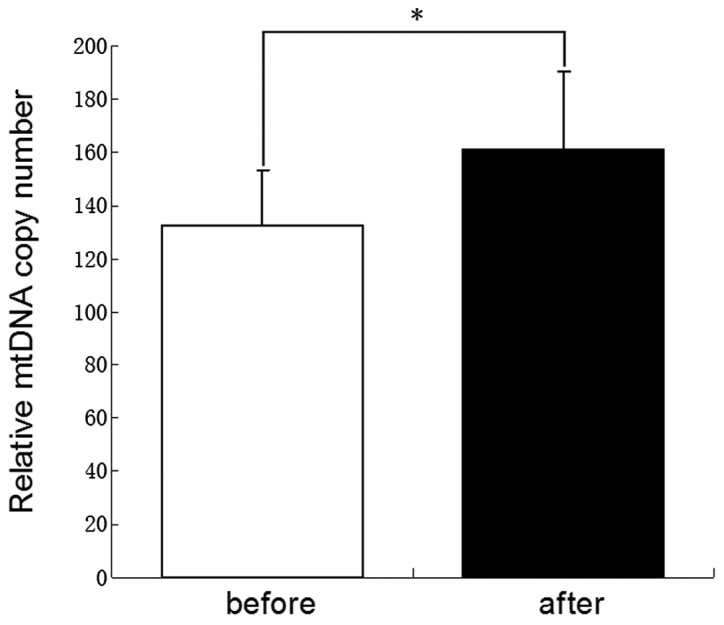
Relative mtDNA copy number of gastric mucosa cells prior to and following 5-Aza treatment. (^*^P<0.05). mtDNA, mitochondrial DNA; 5-Aza, 5-Aza-2′-deoxycytidine.

Prior to and following the treatment with 5-Aza, the primary rat gastric mucosal cells were collected for PCR analysis of the mtDNA copy number. As shown in Fig. 3, the mtDNA copy number of rat gastric mucosal cells was increased significantly subsequent to being treated with 5-Aza (P<0.05).

## Discussion

A growing number of experiments have revealed that mtDNA content variations may affect the behaviors of malignant cells, including cell growth, apoptosis, anticancer drug sensitivity and the invasive and metastatic potentials (10). To date, the characterization of mtDNA by utilizing the power of quantitative PCR assays has revealed the quantitative abnormalities of the mtDNA content in a multitude of human cancers. The alterations in the mtDNA copy number in tumor specimens are usually retained within a relatively stable range in comparison with those in the adjacent non-cancerous tissues (11). The mtDNA content increases in a large number of malignant tumors, including acute lymphoblastic leukemia (12), colorectal carcinoma (13,14) and esophageal squamous cell carcinoma (15,16–18), but decreases in numerous other tumors, including breast cancer (19), hepatocellular carcinoma (20) and Ewing’s sarcoma (21–23). Several studies in colorectal carcinoma (24,25), breast cancer (25) and lung cancer (26) reported the positive association between an increased mtDNA content in peripheral blood specimens and an elevated cancer risk. Further studies on the epigenetic alterations of mtDNA and its downstream products are beneficial to understanding the function of mtDNA in malignancy (27,28).

In the present study, quantitative PCR was performed to measure the relative mtDNA copy number of the tumor and corresponding non-cancerous tissues. Fig. 1A and B show the tendencies of the relative mtDNA copy number between the tumor and corresponding non-cancerous tissues in gastric cancer. The average mtDNA copy numbers of the tumor and corresponding non-cancerous tissues exhibited statistically significant differences.

Furthermore, the mtDNA copy numbers of the tumor and corresponding non-cancerous tissues were compared individually, between males and females. No significant difference was observed between gender. Thus, it appeared that there was no gender effect on the mtDNA copy number in gastric cancer (Fig. 1C).

Advanced gastric cancer is classified into Borrmann types I–IV, with an increasing degree of malignancy (29). Gastric cancers of Borrmann types I and type II are well-defined tumors, while type III and IV are ill-defined tumors with little or no gland-forming capability. The majority of the patients that have tumors of types III and IV have a poor prognosis and low 5-year survival rates following gastric resection (30). In nasal polyp tissue, Park *et al* reported that the change in the mtDNA copy number is correlated with tumor progression (31). The correlation between the relative mtDNA copy number and clinicopathological stages was also analyzed in the present study. Fig. 2 shows that the differences in the relative mtDNA copy numbers between the tumor tissues and the corresponding non-cancerous tissues of stages III and IV are more evident than those of stages I and II. This result is consistent with the phenomenon of the ‘Warburg effect’, which indicates that cancer cells exhibit an enhanced generation of ATP mainly by glycolysis, but not by oxidative phosphorylation (32).

However, mtDNA also contains a non-coding region called the D-loop, which controls the replication and transcription of mtDNA (15,33). Our previous study on colorectal cancer showed that the demethylation of the D-loop is an early molecular event in colorectal cancer. The demethylation of the D-loop may also have an effect on the expression of the ND1 gene, which is encoded by mtDNA (28). In the present study, a demethylation experiment was performed to determine whether the change of mtDNA copy number was associated with D-loop demethylation in gastric cancer. The results (Fig. 3) revealed that following the demethylation treatment, the mtDNA content increased in the gastric cells, suggesting that demethylation of the D-loop may be one of the mechanisms that leads to a decrease in the mtDNA content of gastric cancer cells. However, further studies are required to confirm this hypothesis.

In conclusion, the present study shows that the mtDNA copy number decreases in gastric cancer. This decrease is particularly notable in ill-defined tumors of clinicopathological stages III and IV. The decreased mtDNA copy number is a late molecular event during the progression of gastric cancer. This finding may be used to determine the clinicopathological stage of gastric cancer.

## Figures and Tables

**Table I:** Clinical data of 76 gastric cancer patients.

Characteristic	Gastric cancer patients, n (%)	Age, years (range)
Gender		
Male	47 (61.8)	50 (27–71)
Female	29 (38.2)	53 (34–73)
Clinicopathological stage		
Male		
I	5 (6.6)	49 (27–69)
II	9 (11.8)	47 (37–70)
III	14 (18.4)	52 (28–71)
IV	19 (25.0)	51 (37–70)
Female		
I	4 (5.3)	50 (41–63)
II	6 (7.9)	48 (34–61)
III	10 (13.2)	56 (41–70)
IV	9 (11.8)	54 (39–73)

**Table II:** Relative mtDNA copy number of the various clinicopathological stages.

Tissue type	Stage			

	I	II	III	IV
TT	111±20.89	104.38±26.79	92.85±26.71	85.9±29.34
CNT	120.2±20.62	109.9±22.43	113.10±23.17	108.66±21.05

Data are presented as the mean μ standard deviation. mtDNA, mitochondrial DNA; TT, tumor tissue; CNT, corresponding non-cancerous tissue.

**Table III:** Average relative mtDNA copy number of groups 1 (stages I and II) and 2 (stages III and IV).

Tissue type	Group 1	Group 2
TT	106.86±24.49^a^	89.11±28.10
CNT	113.76±21.91^b^	110.71±21.95

Data are presented as the mean ± standard deviation. mtDNA, mitochondrial DNA; TT, tumor tissue; CNT, corresponding non-cancerous tissue.

a P<0.01 between groups 1 and 2.

b P>0.05 between groups 1 and 2.
